# A Comparative Study on the General and Otolaryngological Manifestations of COVID-19 in the Hospitalized Population of the Telangana Region During the First and Second Waves

**DOI:** 10.1055/s-0043-1777419

**Published:** 2024-02-05

**Authors:** Uma Pokala, Shilpa Potnuru, Sasikala Kanapalli, Agni Vishnu Sailesh, Naveen P.

**Affiliations:** 1 Department of ENT, Mamata Medical College, Khammam, Telangana, India; 2 Department of ENT, NRI Institute of Medical Sciences, Vizag, AP, India; 3 Department of Obstetrics & Gynecology, Vishwabharathi Medical College, Kurnool, Andhra Pradesh, India; 4 Department of Pharmacology, Mamata Medical College, Khammam, Telangana, India

**Keywords:** COVID-19, SARS-CoV-2, anosmia, dysgeusia

## Abstract

**Introduction** Some common symptoms of coronavirus disease 2019 (COVID-19) are fever, cough, and shortness of breath. But ear, nose, and throat (ENT) manifestations such as loss of smell and taste are also very common.

**Objectives** To compare the general and otorhinolaryngological manifestations of COVID-19 and to compare the treatments given and mortality rate during its two waves.

**Methods** This retrospective study was conducted on severe acute respiratory syndrome coronavirus 2 (SARS-CoV-2) cases in a tertiary care teaching hospital. Six hundred patients were included in the 1st wave sample and 516 were in the 2nd wave sample. The data collected included demographics, comorbidities, general, and ENT symptoms, need for ventilatory support, oxygen therapy, and mortality for both the waves.

**Results** Fever, malaise, and myalgia were more frequently presented in the first wave than in the second, whereas shortness of breath was more common in the second wave. In the second wave, a significant increase in anosmia cases was reported, whereas sore throat, nasal obstruction, dysphagia, nasal discharge, and sneezing were significantly reduced compared with the first wave ( *p* < 0.001). The case fatality rate increased from 11.33 to 21.55% ( *p* < 0.001) from the 1 ^st^ to the 2 ^nd^ wave. The patients who died in the second wave were younger than those in the first wave. Two doses of vaccination showed protection from the death over those not vaccinated and those who only received one dose ( *p* < 0.05).

**Conclusion** Ear, nose, and throat (ENT) manifestations are very common along with the general symptoms. As anosmia and dysgeusia are early presenting symptoms in COVID-19 patients, all physicians should screen patients for ENT symptoms.

## Introduction

Coronavirus is a single-stranded RNA virus, with a diameter of ∼ 60 to 140 nm. 1 Coronavirus disease 2019 (COVID-19) was confirmed as a pandemic in March 2020 by the World Health Organization (WHO). 2 The first COVID-19 case was detected in India on January 30, 2020, and the number of cases gradually increased within a few months. In India, the first wave began from April 2020 to the end of that year, and the second wave began in March, peaked in April, and ended by August 2021. Though the commonest symptoms are lower respiratory tract symptoms, such as fever, cough, and shortness of breath, ear, nose, and throat (ENT) manifestations like sore throat, nasal congestion, olfactory dysfunction (OD), and gustatory dysfunction (GD) also are common. 3 Olfactory dysfunction and GD are the only manifestations in asymptomatic individuals. 4 Olfactory dysfunction has a sudden onset, transient duration, and the recovery is very fast, with symptoms mostly being nasal-related without congestion or discharge. 5 Though the etiopathogenesis of OD is not clear, damage to the olfactory neurons, cortex, and epithelium are the possible mechanisms. The prevalence of OD was 47.85% worldwide. 6 In India, there was a shortage of hospital beds and oxygen cylinders, especially in wave 2, leading to a higher number of deaths. 7

Though several studies had been conducted globally, studies on the Indian population are very few. The aim of our study is to compare general and ENT manifestations of the first and second waves. We compared general symptoms like fever, cough, shortness of breath, and ENT symptoms like olfactory and gustatory dysfunction, sore throat, nasal discharge, and nasal obstruction between the two waves. Otolaryngologists should be aware of COVID-19 presenting symptoms related to their specialty as the first death COVID-19-related death was an otolaryngologist. 8 We compared the differences in the treatment given and mortality rate in the two waves. We have also shown the effect of vaccination on the death rate during the second wave. We hope this study will help and guide otolaryngologists to initiate early treatment to prevent long-term complications in upcoming waves if there are any.

## Material and Methods

This retrospective study was conducted on hospitalized cases of severe acute respiratory syndrome coronavirus 2 (SARS-CoV-2) infection in a tertiary care hospital in the Telangana state in India after getting approval from an institutional human ethics committee (IEC/IRB No: 86/21). The period of our study during the first wave was from August 2020 to January 2021, and the second wave was from April to August 2021. Consent was obtained from every patient before recruiting them into the study, and confidentiality was maintained regarding patient details.

### Inclusion Criteria

All COVID-19 hospitalized symptomatic patients with positive reverse transcription polymerase chain reaction (RT-PCR) report.

### Exclusion Criteria

Pregnancy and lactating women.

Malignant COVID-19-positive patients.

Patients with no response to phone call for further collection of data.

The study data was taken from the medical records of patients retrospectively, regarding demographic and clinical details during their hospital stay. Information regarding their death status was taken through telephonic conversation. Death within the 1 month after discharge from our hospital was only considered as death in our study. The data collected included demographics, comorbidities, general and ENT symptoms, need for ventilatory support, oxygen therapy, and mortality of both the waves.

### Sample Size

Six hundred patients were included in the first wave sample and 515 were in the second wave sample.

### Statistical Analysis

The collected data was tabulated in a Microsoft Excel sheet (Microsoft Corp., Redmond, WA, USA) and analyzed. For numerical data, means with standard deviation were used, and for categorical data, proportions were expressed. To measure the statistical significance, the Z test was performed for numerical data and the Chi-squared test **(χ2** ) for categorical data. Multivariate logistic regression analysis was performed to identify the mortality predictors. Age, sex, presence or absence of comorbidities, olfactory and gustatory dysfunction, fever, cough, shortness of breath, need for oxygen therapy, and ventilation support were taken as independent variables and death as a dependent variable. A *p* -value < 0.05 was considered statistically significant. The IBM SPSS Statistics for Windows, version 26.0 software (IBM Corp., Armonk, NY, USA) was used for all calculations.

## Results

In the Telangana state, the first wave peaked in the last quarter of 2020 and was followed by a progressive decrease, with very few patients being admitted in January 2021. The number of cases again fluctuated upward from April to August 2021. The number of patients admitted was 629 in the 1st wave and 557 in the 2nd one. As per our inclusion criteria, 600 patients were taken into the 1st wave sample and 515 in the 2nd wave sample. Those in the 2nd wave were significantly younger (50.91 ± 14.64 versus 52.57 ± 15.62 years; *p* < 0.001). In both waves, there were more male than female patients, and the 41 to 60 age group had the highest number of patients in both waves. None of the patients were vaccinated in the first wave as there was no availability of any COVID vaccines then, but in the second wave, 13.39% had received their 1st dose of vaccination, and 5.24% had received both doses of vaccine by the end of August 2021. The most relevant comorbidities were hypertension, type 2 diabetes mellitus, asthma, and coronary heart disease (CAD) ( Table 1 ). The most common general symptoms in both waves were fever and cough, followed by shortness of breath (SoB), malaise, headache, and gastrointestinal symptoms. The most common ENT symptoms were OD (anosmia) and GD (dysgeusia) followed by sore throat, nasal obstruction, nasal discharge, and sneezing. Fever, malaise, and myalgia were more frequently presented during more often in the first wave than in the second, whereas SoB was more common in the second wave than in the first ( *p* < 0.001). In the second wave, a significant increase in anosmia cases was reported whereas sore throat, nasal obstruction, dysphagia, nasal discharge, and sneezing were significantly reduced ( *p* < 0.001). During the second wave, we observed blackish discoloration over the face (nasal bridge, infraorbital margin, around the nose or eyes) in 8 (1.55%) patients ( Table 2 ). Both OD and GD were observed more frequently in males in both the waves. Significantly increased frequency of OD and GD were observed in the 41 to 60 years age group and lower frequency of OD and GD was observed in the 61 to 80 years age group during the second wave. Significant reductions in OD and GD were observed in the vegetarian group during the second wave ( *p* < 0.001). No significant association was observed between comorbidities and chances of having OD and GD. The duration of OD and GD in most of the patients was 5 to 8 days in both waves. No significant change in the duration of OD and GD was observed between the two waves ( *p* > 0.05) ( Table 3 ). In relation to OD with GD, patients who reported to have OD were also having GD and vice versa during the first wave ( *p* < 0.0001) ( Table 4 ). The second wave presented almost the same results as first wave, but since all were having either OD or GD, we could not measure the statistical significance ( Table 5 ).

**Table 1:** Demographic and clinical characteristics of patients with COVID- 19

Feature	First wave (n = 600)	Second wave (n = 515)	*P* -value
Male	415 (69.16%)	355 (68.93%)	0.98
Female	185 (30.83%)	160 (31.06%)	
**Age**			
10–20 yrs	9 (1.5%)	7 (1.35)	0.95
21–40 yrs	137 (22.83%)	127 (24.66%)	0.51
41–60 yrs	259 (43.16%)	254 (49.32%)	< 0.05
61–80 yrs	182 (30.33%)	115 (22.33%)	< 0.01
> 80 yrs	13 (2.16%)	12 (2.33%)	0.98
**Wearing mask habit**			
Always	293 (48.83%0	417 (80.97%)	< 0.001
Occasionally	196 (32.66%)	71 (13.78%)	< 0.001
Never	111 (18.5%)	27 (5.24%)	< 0.001
**Vaccination status**			
Dose I	0 (0%)	96 (13.39%)	NA
Dose II	0 (0%)	27 (5.24%)	
**Dietary habits**			< 0.001
Vegetarian	145 (24.16%)	75 (14.56%)	
Mixed	455 (75.83%)	440 (85.43%)	
**Smoking habit**			0.91
Smokers	34 (5.66%)	29 (5.63%)	
Non-smokers	566 (93.33%)	486 (94.36%)	
**Comorbidities**			
Hypertension	231 (38.5%)	179 (34.75%)	0.19
Diabetes	234 (39%)	196 (38.05%)	0.74
Asthma	25 (4.16%)	19 (3.16%)	0.68
Hypothyroidism	25 (4.16%)	33 (6.40%)	0.12
CAD	18 (3%)	12 (2.33%)	0.61

Abbreviation: CAD, coronary artery disease.

**Table 2:** Comparison of general symptoms during both waves

Feature	First wave (n = 600)	Second wave (n = 515)	*P* -value
Fever	547 (91.16%)	397 (76.5%)	< 0.001
Cough	420 (70%)	361 (70.1%)	0.97
SOB	148 (24.66%)	232 (45.05%)	< 0.001
Malaise	398 (66.33%)	170 (33%)	< 0.001
Myalgia	205 (34.16%)	75 (14.56%)	< 0.001
Headache	79 (13.16%)	65 (12.62%)	0.85
Chest pain	0 (0%)	4 (0.78%)	NA
Vomiting	35 (5.83%)	31 (6.02%)	1
Diarrhea	36 (6%)	25 (4.85%)	0.47
Abdominal pain	21 (3.5%)	8 (1.55%)	0.06
**ENT symptoms**			
Anosmia	263 (43.83%)	273 (53%)	< 0.001
Dysgeusia	266 (44.33%)	255 (49.51%)	0.09
Sore throat	101 (16.83%)	34 (6.60%)	< 0.001
Nasal obstruction	100 (16.66%)	41 (7.96%)	< 0.001
Giddiness	4 (0.66%)	0 (0%)	< 0.001
Dysphagia	42 (7%)	8 (1.55%)	< 0.001
Nasal discharge	112 (18.66%)	51 (9.90%)	< 0.001
Sneezing	38 (6.33%)	6 (1.17%)	< 0.001
Nil	232 (38.66%)	193 (37.47%)	< 0.001
Blackish discoloration around nose	0 (0%)	8 (1.55%)	NA

Abbreviations: ENT, ear, nose, and throat; SoB, shortness of breath.

**Table 3:** Prevalence of olfactory and gustatory dysfunction during both the waves

OD				GD		
Feature	First wave (n = 263)	Second wave (n = 273)	*P* -value	First wave (n = 266)	Second wave (n = 255)	*P* -value
**Gender**						
Male	169 (64.25%)	182 (66.66%)	0.62	171 (64.3%)	174 (68.24%)	0.38
Female	94 (35.74%)	91 (33.33%)		95 (35.7%)	81 (31.76%)	
**Age group (years)**						
10–20	1 (0.76%)	3 (1.09%)	0.96	2 (0.75%)	2 (0.78%)	0.64
21–40	72 (27.37%)	74 (27.10%)	0.97	74 (27.81%)	70 (27.45%)	1
41–60	110 (41.82%)	148 (54.21%)	< 0.01	108 (40.6%)	137 (53.72%)	< 0.01
61–80	79 (30.03%)	42 (15.38%)	< 0.001	81 (30.45%)	42 (16.47%)	< 0.001
> 80	1 (0.38%)	6 (2.19%)	0.14	1 (0.38%)	4 (1.56%)	0.34
**Smoking habits**			0.31			0.16
Smokers	12 (4.56%)	19 (6.95%)		12 (4.52%)	20 (7.84%)	
Nonsmokers	251 (95.43%)	254 (93.04%)		254 (95.48%)	235 (92.16%)	
**Diet**			< 0.001			< 0.001
Vegetarian	63 (23.95%)	27 (9.89%)		64 (24.06%)	27 (10.6%)	
Mixed	200 (76.04%)	246 (90.10%)		202 (75.94%)	228 (89.4%)	
**Comorbidities**						
DM	109 (41.44%)	94 (34.43%)	0.11	111 (41.72%)	87 (34.11%)	0.08
HTN	107 (40.68%)	89 (32.60%)	0.06	107 (40.22%)	81 (31.76%)	0.05
**Duration**						
0–4 days	52 (19.77%)	65 (23.80%)	0.3	54 (20.30%)	56 (21.96%)	0.79
5–8 days	86 (32.69%)	103 (37.72%)	0.25	87 (32.70%)	99 (38.82%)	0.17
9–14 days	50 (19.01%)	43 (15.75%)	0.37	47 (17.66%)	39 (15.29%)	0.54
15 days–1 month	63 (23.95%)	51 (18.68%)	0.16	66 (24.81%)	52 (20.39%)	0.27
> 1 month	12 (4.56%)	11 (4.02%)	0.92	12 (4.51%)	9 (3.52%)	0.72

Abbreviations: DM, diabetes mellitus; GD, gustatory dysfunction; HTN, hypertension; OD, olfactory dysfunction.

**Table 4:** Correlation between olfactory and gustatory dysfunction during the first wave

	GD (n = 266)			*P* -value
		yes	no	
OD (n = 263)	Yes	243	20	< 0.0001
	No	23	71	

Abbreviations: GD, gustatory dysfunction; OD, olfactory dysfunction.

**Table 5:** Correlation between olfactory and gustatory dysfunction during the second wave

	GD (n = 255)			*P* -value
		yes	no	
OD (n = 273)	Yes	249	24	Not possible
	No	6	0	

Abbreviations: GD, gustatory dysfunction; OD, olfactory dysfunction.

We also evaluated the differences in treatments between the two groups of patients. Subjects from the second wave were treated more often with assisted ventilation and oxygen therapy. A significant increase in the need for oxygen therapy was observed during the second wave ( *p* < 0.01). The use of intravenous corticosteroids was significantly increased during the second wave whereas the use of oral steroids was significantly reduced ( *p* < 0.01) ( Table 6 ). Finally, we wanted to evaluate the comparison of deaths in both waves, and a total of 68 deaths occurred during the first wave and 111 during the second wave, so the case fatality rate increased from 11.33 to 21.55% ( *p* < 0.001). Interestingly, the death rate was higher in females in both the waves, but it was not statistically significant ( *p* > 0.05). During the first wave, more deaths (48.52%) were seen in the 61 to 80 age group, but in the second wave, more deaths (45.94%) were observed in the 41 to 60 years age group. The patients who died in the second wave were younger than those in the first wave (57.45 ± 13.96 versus 61.85 ± 16.20 years; *p* < 0.05). A significantly lower number of deaths were observed in vegetarians in both waves ( Table 7 ). We found some differences in the risk factors associated with mortality between the two waves. As per multiple regression analysis OD, GD, SOB, oxygen therapy, and assisted ventilation were associated with mortality during the first wave, while old age, SoB, myalgia, oxygen therapy, and assisted ventilation were associated with higher mortality during the second wave ( Tables 8 and 9 ). A percentage of 79.27% of deaths in the second wave were in non-vaccinated patients and 20.7% in vaccinated patients ( Table 7 ). Two doses of vaccination showed protection from death compared with not being vaccinated ( *p* < 0.05) and one dose of vaccination only ( *p* < 0.05) ( Tables 10 and 11 ).

**Table 6:** Comparison of the treatments given during the first and second waves

Feature	First wave	Second wave	*P* -value
	(n = 600)	(n = 515)	
**Corticosteroids**	600 (100%)	501 (97.3%)	
Oral and IV	370 (61.66%)	379(75.64%)	< 0.001
Oral	227 (37.83%)	65 (12.62%)	< 0.001
IV	03 (0.5%)	57 (11.37%)	< 0.001
**Oxygen therapy**			< 0.001
Given	316 (52.66%)	402 (78%)	
Not given	284 (47.33%)	113 (21.94%)	
**Assisted ventilation**	37 (6.16%)	38 (7.37%)	0.49
BPAP	9 (24.32%)	8 (21.05%)	
CPAP	28 (75.67%)	30 (78.94%)	
Not given	563 (93.83%)	477 (92.62%)	

Abbreviations: BPAP, bi-level positive airway pressure; CPAP, continuous positive airway pressure.

**Table 7:** Comparison of the deaths during both waves

Feature	First wave (n = 68)	Second wave (n = 111)	*P* -value
**No of deaths**	68 (11.33%)	111 (21.55%)	< 0.001
**Gender**			0.52
Male	17 (25%)	34 (30.63%)	
Female	51 (75%)	77 (69.36%)	
**Age group**			
10–20	1 (1.47%)	1 (0.90%)	0.7
21–40	6 (8.82%)	12 (10.81%)	0.86
41–60	21 (30.88%)	51 (45.94%)	0.04
61–80	33 (48.52%)	44 (39.63%)	0.31
> 80	7 (10.29%)	3 (2.70%)	0.07
**Vaccination status**			NA
I dose	0 (0%)	22 (19.81%)	
Doses I & II	0 (0%)	1 (0.9%)	
Not vaccinated	68 (100%)	88 (79.27%)	
**Diet**			0.71
Veg	12 (17.64%)	16 (14.41%)	
Mixed	56 (82.35)	95 (85.6%)	
**ENT symptoms**			
Anosmia	17 (25%)	47 (42.34%)	< 0.05
Dysgeusia	14 (20.6%)	45 (40.54%)	0.12
**Comorbidities**			
Hypertension	36 (52.9%)	49 (44.14%)	0.32
Diabetes	31 (45.6%)	53 (47.74%)	0.9
Asthma	5 (7.35%)	7 (6.30%)	0.97
Hypothyroidism	3 (4.41%)	6 (5.40%)	0.95
CAD	7 (10.3%)	3 (2.70%)	0.07

Abbreviation: CAD, coronary artery disease; ENT, ear, nose, and throat.

**Table 8:** Logistic regression analysis of the risk factors association with deaths for patients during the first wave of COVID-19

Variables in the equation									
		B	SE	Wald	df	Sig.	Exp (B)	95% CI for EXP (B)	
								Lower	Upper
Step 1 ^st^	Age group	0.727	0.511	2.023	1	0.155	2.070	0.760	5.638
	Sex	−0.376	0.496	0.574	1	0.449	0.687	0.260	1.816
	Smoking	−0.215	0.760	0.080	1	0.777	0.807	0.182	3.576
	Diet	0.303	0.242	1.562	1	0.211	1.353	0.842	2.175
	Comorbidities	−1.050	0.575	3.333	1	0.068	0.350	0.113	1.080
	Anosmia (OD)	−2.460	0.950	6.703	1	0.010	0.085	0.013	0.550
	Dysgeusia (GD)	4.839	2.116	5.231	1	0.022	126.325	1.998	7,985.218
	OD and GD	−1.246	2.240	0.309	1	0.578	0.288	0.004	23.217
	Nasal obstruction	−0.249	0.744	0.112	1	0.738	.779	0.181	3.353
	Nasal discharge	0.080	0.655	0.015	1	0.903	1.083	0.300	3.910
	Sore throat	0.267	0.634	0.177	1	0.674	1.306	0.377	4.521
	Fever	−0.336	0.777	0.187	1	0.665	0.714	0.156	3.278
	Cough	0.114	0.498	0.053	1	0.818	1.121	0.423	2.973
	SoB	−1.929	0.703	7.527	1	0.006	0.145	0.037	0.576
	Malaise	0.292	0.434	0.455	1	0.500	1.340	0.573	3.135
	Myalgia	0.472	0.500	0.892	1	0.345	1.603	0.602	4.268
	Oxygen therapy	−3.298	1.109	8.853	1	0.003	0.037	0.004	0.324
	Assisted ventilation	−6.084	1.127	29.120	1	0.000	0.002	0.000	0.021
	Constant	12.749	4.003	10.145	1	0.001	344153.867		

Abbreviations: COVID-19, coronavirus disease 2019; GD, gustatory dysfunction; OD, olfactory dysfunction; SoB, shortness of breath.

a. Variable(s) entered on step 1: age group, sex, smoking, diet, comorbidities, anosmia, dysgeusia, OD and GD, nasal obstruction, nasal discharge, sore throat, fever, cough, SoB, malaise, myalgia, oxygen therapy, assisted ventilation.

**Table 9:** Logistic regression analysis of the risk factors association with deaths for patients during the second wave of COVID-19

Variables in the equation wave 2									
		B	SE	Wald	df	Sig.	Exp (B)	95% CI for EXP(B)	
								Lower	Upper
Step 1 ^st^	Age group	1.090	0.334	10.635	1	0.001	2.974	1.545	5.725
	M/F	−0.101	0.288	0.122	1	0.727	0.904	0.514	1.591
	Smoking status	−0.535	0.474	1.272	1	0.259	0.586	0.231	1.484
	Veg/Mixed	−0.201	0.362	0.308	1	.579	0.818	0.403	1.662
	Comorbidities	−0.358	0.281	1.622	1	0.203	0.699	0.403	1.213
	Vaccination status	0.483	0.316	2.338	1	0.126	1.621	0.873	3.010
	OD	0.491	0.620	0.626	1	0.429	1.634	0.484	5.510
	GD	−0.364	1.107	0.108	1	0.743	0.695	0.079	6.092
	OD and GD	0.113	1.128	0.010	1	0.920	1.120	0.123	10.221
	Nasal obstruction	0.598	0.563	1.129	1	0.288	1.819	0.603	5.482
	Nasal discharge	−0.699	0.474	2.173	1	0.140	0.497	0.196	1.259
	Sore throat	−0.011	0.575	0.000	1	0.985	0.989	0.320	3.056
	Fever	0.002	0.301	0.000	1	0.995	1.002	0.556	1.806
	Cough	−0.169	0.287	0.349	1	0.555	0.844	0.481	1.481
	SoB	−0.855	0.307	7.781	1	0.005	0.425	0.233	0.775
	Malaise	−0.284	0.266	1.135	1	0.287	0.753	0.447	1.269
	Myalgia	1.163	0.492	5.589	1	0.018	3.199	1.220	8.387
	Oxygen therapy	−1.763	0.552	10.187	1	0.001	0.172	0.058	0.506
	Assisted ventilation	−2.080	0.436	22.774	1	0.000	0.125	0.053	0.294
	Constant	3.222	2.611	1.523	1	0.217	25.076		

Abbreviations: COVID-19, coronavirus disease 2019; GD, gustatory dysfunction; OD, olfactory dysfunction; SoB, shortness of breath.

a. Variable(s) entered on step 1: age group, m/f, smoking status, veg/mixed, comorbidities, vaccination status, OD, GD, OD and GD, nasal obstruction, nasal discharge, sore throat, fever, cough, SOB, malaise, myalgia, oxygen therapy, CPAP/BPAP.

**Table 10:** Effect of 2 doses of vaccination over not vaccination during wave 2

	Deaths	No death	*p* -value
Not vaccinated	88	304	0.03
Vaccinated with 2 doses	1	26	

**Table 11:** Effect of 2 doses of vaccination over 1 dose of vaccination during wave 2

	Deaths	No death	*p* -value
Vaccinated with 1 dose	22	74	0.04
Vaccinated with 2 doses	1	26	

## Discussion

During the second wave, more younger adults were admitted than in the first wave, and more old-age patients were admitted during the first wave. The reason could be the imprudent behavior of young adults toward social distancing, especially during the second wave, after a prolonged period of lockdown. The number of OD and GD cases was higher in the second wave. The case fatality rate was higher in the second wave, which could be attributed to the emergence of a SARS-CoV-2 new variant at the time of the second wave in India.

In both waves, more males were admitted than females, the reason behind this may be because of the strong innate and adaptive immune responses of females. 9 The most common comorbidities were hypertension and diabetes in both waves. Similar findings were shown by Iftimie et al., Haung et al., and Wang et al. 9 10 11 In the present study, the most common ENT manifestations were OD and GD followed by nasal discharge, sore throat, and nasal obstruction. In the first wave, GD cases were higher whereas OD cases were higher in the second wave. Lechien et al. also reported GD in 89% of their cases ( *n* = 342/417) and OD disorders in 86% of cases ( *n* = 357/417). 12 Similar findings were also reported in a study conducted in California (OD in 68% and GD in 71%). 13 The pathophysiology of OD is unclear. Though some studies suggested the expression of ACE2 receptors on the subtentacular and horizontal basal cells of the olfactory mucosa, 14 little is known about the target cells of SARS-CoV-2 and whether the virus can attack sensory cells or the olfactory bulb directly. A possible mechanism for GD is the presence of ACE2 receptors on the epithelium of taste buds and salivary glands, which is targeted by SARS-CoV-2.

In the present study, a strong association between OD and GD was observed in both waves. Similar results were reported by Klopfenstein et al. (anosmia was associated with dysgeusia in 85% of cases). 15 Most of the patients recovered from OD and GD within 8 days in both the waves. Lechien et al. also reported that 73% of patients recovered completely from OD and GD within the first 8 days. 12 Short-lived presentation of OD and GD could be rapid regeneration of damaged epithelium after viral clearance and early development of an antibody response, which results in less severe symptomatology that requires hospitalization.

In the comparison of the treatment of both waves, the routes of administration of corticosteroids were shifted from oral to intra venous route from wave 1 to wave 2. The need for oxygen therapy was increased from wave 1 to wave 2 ( *p* < 0.001). The second wave of theCOVID-19 pandemic in India was brutal, completely overwhelming the public health system, especially hospital care because of a shortage in oxygen supply. This finding contrasted with the study by Oladunjoye et al., in which there was no significant difference in the usage of oxygen between the two waves (18.7% vs 18.4%). 16 A noteworthy finding in our study was the case fatality rate, which was significantly higher ( *p* < 0.001) in the second wave than in the first. This finding was in unison with the study conducted in India by Vohra et al., in which the mortality was higher in the second wave ( *n* = 537) than in the first ( *n* = 172), 17 but in contrast to the study conducted in the USA by Oladunjoye et al., in which mortality was higher in the first wave (23.2% vs 12.3%, *p* < 0.001). 16 During the second wave, the death rate was reduced in the patients who had received 2 doses of vaccine in comparison to patients who had not been vaccinated ( *p* < 0.05). Two doses of vaccination significantly reduced mortality when compared with one dose of vaccination ( *p* < 0.05).

## Conclusion

The results of our study showed that ENT manifestations are very common along with general symptoms. A relatively young age group was affected more during the second wave. As OD and GD are early presenting symptoms in COVID-19 patients, otolaryngologists and all physicians should have aware of this to take necessary action to diagnose and contain the spreading of the disease. Further prospective studies are needed to study the ENT manifestations in detail, post-COVID complications, if any, and the efficacy of vaccination.

## Limitations

The primary limitation was that the study was conducted in a single institution and its duration was short. Some important data was collected through telephone conversations, which can lead to recall bias. As patient follow-up was not possible during the pandemic, we missed registering post-COVID permanent sequalae, if any. During the study period, fewer patients were vaccinated with 2 doses.
